# Evaluation of Yeasts and Yeast Products in Larval and Adult Diets for the Oriental Fruit Fly, *Bactrocera dorsalis*, and Adult Diets for the Medfly, *Ceratitis capitata*, and the Melon Fly, *Bactrocera curcurbitae*

**DOI:** 10.1673/031.009.2301

**Published:** 2009-05-22

**Authors:** Chiou Ling Chang

**Affiliations:** U. S. Pacific Basin Agricultural Research Center, USDA-ARS, 2727 Woodlawn Drive, Honolulu, Hawaii 96822

**Keywords:** fruit fly rearing, yeast products, liquid diet, protein resource

## Abstract

Several yeasts and yeast products were tested in adult diets for the medfly *Ceratitis capitata* (Wiedemann), oriental fruit fly *Bactrocera dorsalis* (Hendel), and melon fly, *Bactrocera curcurbitae* (Coquillett) (Diptera: Tephritidae) and in larval liquid diet for mass-rearing *B. dorsalis*. Three hydrolyzed brewer's yeasts (FNILS65, FNI200 and FNI210), one glutamine enriched yeast (GSH), one vitamin-enriched yeast (RDA500), Korea yeast, whole cell yeasts, and combinations of them were evaluated. Adult flies fed on a diet with FNI210FNI210 + GSH and RDA500 produced the highest number of eggs in all three tested fruit fly species. However, no significant difference was seen in egg hatch from flies fed on these diets with yeast in comparison to the control standard diet. When these yeasts were incorporated into a larval liquid diet with wheat germ oil, FNI200 and FNIL65 showed significantly higher pupal recovery than those from FNI210 and better adult flying and mating than those from Korea yeast. Glutamine enriched yeast enhanced fly performance, especially with FNI200 + GSH and FNILS65 + GSH, but not vitamin enriched yeast. Among the larvae reared with FNI200 + GSH, FNILS65 + GSH and torula yeast, those reared in FNILS65 + GSH diet with wheat germ oil developed the best. In order to select the most cost-effective yeast for liquid diet, FNILS65 + GSH and wheat germ oil was combined with whole cell yeast (LBI2240 series) and compared to the control diet (conventional mill feed diet currently used in the rearing facility). A ratio of 3:1 of LBI2240 and FNILS65 + wheat germ oil was selected as the most effective yeast for oriental fruit fly liquid larval diet based on cost and performance parameters.

## Introduction

Production of low cost but high quality sterile insects is a goal for successful sterile insect technique programs. Yeast products are the main nutritional component in the diet used to mass-rear the adults and larvae of fruit flies in these programs (Schroeder et al. 1972; Cangussu and Zucoloto 1992, 1997; Placido-Silva et al. 1997; Aluja et al. 2001; Rohlfs and Hoffmeister 2005). Brewer's yeast, *Saccharomcyes cerevisiae*, and torula yeast, *Candida utilis* are the two most commonly used yeasts in mass rearing fruitflies.

The high cost of using imported brewer's yeast for larval and adult mass rearing made screening other more cost effective local yeasts a priority. Yeasts such as FNILS65, FNI200, FNI210, LBI2240, GSH, and RDA500 can be obtained conveniently (Lallemand, www.lallemand.com). FNI LS65 is a primary grown yeast extract powder, i.e. it is not grown on grain. FNI200 is a brewer's yeast peptone powder while FNI210 is a distilling yeast peptone powder. Both FNI200 and FNI210 contain all the essential microbial growth factors for both fruit fly such as high level of amino nitrogen, peptides, vitamins and minerals and are economical and good alternatives used for many types of fermentations including cultures and bioremediation. LBI2240 is a debittered brewer's yeast that has been debittered without the use of chemicals and deactivated by being heated and roller dried. LBI2240 is a whole cell yeast while FNILS65, FNI200, and FNI210 are hydrolyzed yeasts.

A “liquid” larval diet has been developed that alleviates the need for a bulking agent, to replace the currently used “USDA standard” artificial diet for fruit flies, which primarily consists of sucrose, protein and a bulking agent (mill feed) (Tanaka et al. 1969). If the liquid diet is incorporated into a mass-rearing facility, the costs associated with the disposal of the standard diet can be reduced, and it saves potential space and lowers environmental impact.

In this study 14 yeasts and yeast products were evaluated for use in adult diets and 21 yeasts and yeast products for larval liquid diet formulation respectively, to determine the best product for cost effective mass rearing. Various biological developmental parameters of the flies were compared after they fed on the experimental diets or standard “millfeed” diet. Brewer's yeast was not used as a control as it showed no significant differences from the millfeed diet (Chang et al. 2006).

## Materials and Methods

### Insects

Larvae of the oriental fruit fly, *Bactrocera dorsalis* (Hendel) (Diptera: Tephritidae), were used to evaluate various yeast products in larval liquid diet; and adults of the medfly, *Ceratitis capitata* (Wiedemann), oriental fruit fly, *B. dorsalis* (Hendel), and melon fruit fly, *B. cucurbitae* (Coquillett) (genetic sexing strain) were used to evaluate various yeast products in adult diets. Eggs collected within an hour of oviposition and pupae collected 2 days after larval collection were provided by the fruit fly rearing unit of Agricultural Research Service, Pacific Basin Agricultural Research Center in Honolulu, Hawaii throughout this study. Both diets and insects were maintained in 25°C, 65% RH and 12: 12 L:D.

### Tested yeast products and chemicals

Yeast products used in this study include four main yeasts, FNILS65, FNI200, FNI210, and LBI2240, glutamine enriched yeast, GSH (G), high B-complex vitamin enriched yeast, RDA500 (R) and their combinations (FNILS65, FNILS65R, FNILS65G, FNILS65GR, FNI200, FNI200R, FNI200G, FNI200GR, FNI210, FNI210R, FNI210G, FNI210GR, LBI2240, LBI2240G, LBI2240FNILS65) provided by Lallemand were evaluated along with Korea yeast (K) which was purchased from Beer Yeast Korea Co., Ltd (www.Beeryeastkorea.com), and torula yeast (T) which was purchased from Borregaard (www.borregaard.com) and used in liquid diets and as standard control diets were also evaluated. Wheat germ oil (W) was used as a nutritional enhancer because our previous work showed that addition of wheat germ oil to a liquid diet would enhance performance (Chang and Vargas 2007). All nutritional information on yeast products used in this study is shown in Table 1. Brewer's yeast was not as a control as it showed no significant differences from the millfeed diet (Chang et al. 2006).

### Adult diets

Five grams of FNILS65, FNI200, or FNI210 each and their nine products, FNILS65G, FNILS65R, FNILS65GR, FNI200G, FNI200R, FNI200GR, FNI210G, FNI210R, FNI210GR and the two control yeasts from ICN Biochemicals (ww.icnbiomed.com) and USB Biochemicals (www.usbweb.com), respectively, were mixed with 15 grams of sucrose to construct a 20 grams of yeast/sugar (1:3) fruit fly adult diet for each adult cage. Ten grams of pupae (equal to approximately 1000 adults) each were set up in each metal adult cage (26 cm × 26 cm × 26 cm) for adult eclosion along with twenty grams each of diet and water. Adults were fed *ad lib*. throughout the experiment. At sexual maturity (11 days for the oriental fruit fly, melon fly, and 7 days for the medfly), an oviposition cup (12 oz, 9 × 7 cm; a 8 oz honey cup with 250 perforated holes) was inserted into each cage for 24 h for 7 days. Eggs collected cumulatively through these seven days were used to calculate the numbers of eggs per female per day (Chang et al, 2006). Each treatment had four cages and was repeated three times from three different batches of flies and the data were analyzed using SAS one-way ANOVA (SAS, 2002). The evaluation parameters for adult diets were egg production and egg hatch.

**Table 1.**
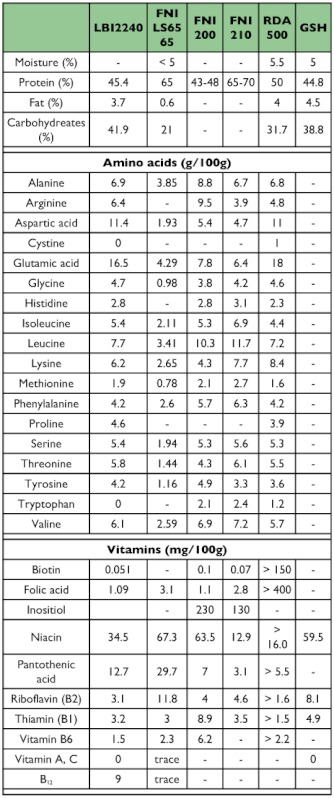
Comparative nutrients from LBI2240, FNI LS65, FNI 200, FNI 210, RDA500, and GSH (Data were provided by Lallemand Bio-Ingredients)

**continued:**
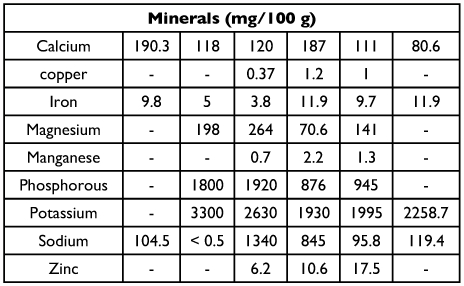

### Larval liquid diet

Larval liquid diet was prepared as described by Chang et al. (2004, 2006). Yeasts including FNILS65, FNI200, FNI210, LBI2240, Korea yeast, torula yeast, and their combinations were substituted (wt: wt) for brewer's yeast in the previous published diet (Chang et al. 2004; 2006). Liquid diet tests were conducted using standard fruit fly mass rearing fiberglass trays (49.7 × 32 × 2.5cm^3^) (Stack and Nest, www.orbiscorporation.com). The diet mixture was formulated by weighing all the dry ingredients and blending them with 1000 ml of distilled water until the ingredients were fully dissolved and homogeneously suspended (approximately 2 min). Two pieces of sponge cloth (15 cm × 10 cm × 0.2 cm and 30 cm × 26 cm × 0.2 cm, Kalle Inc., Flemington, NJ 08822) were used in each larval tray. The sponge was rinsed in cold distilled water and wrung dry. The top and bottom of this sponge cloth have different surface patterns — one is a diamondshaped and the other has grooves. The sponge cloth, with the grooved pattern face up, that served as the primary support matrix for feeding larvae, was laid on the top of hardware net (0.5″ mesh, Tenax Corporation, MD) on the bottom of the tray. One liter of the liquid diet was then poured over the sponge cloth to saturation and any extra liquid was allowed to flow over the bottom of the larval tray below the sponge (which helped maintain the moisture and distribute the food source evenly). This provided a suitable substrate for the developing larvae, especially for the 1^st^ and 2^nd^ instars.

About 37,500 (2.5 ml, approximately 15,000 eggs per ml for the oriental fruit fly) one-hour-old eggs were applied to a piece of sponge (2.5 × 10cm), with diamond-shaped pattern face up, using either a plastic pipette or dispenser. The eggs were sprayed gently with distilled water to spread them evenly across the sponge surface. The sponge was then placed on top of the diet saturated larger sponge. Egg hatch was determined by deducting the number of un-hatched eggs from 100 eggs each on a blotting paper after 72 hours. The average egg hatch was obtained from the mean of four replicates. Upon egg hatch, larvae fed *ad libitum* in the liquid diet at 25°C, 65% RH until larva began to jump out of the larval tray after 8 days. Larvae were collected into water, and were sieved into vermiculite for pupariation. Pupae were sieved from vermiculite and collected daily for four consecutive days. All the tests in this study were carried out at 25 °C, 65% RH, and 12:12 L:D.

**Table 2.**
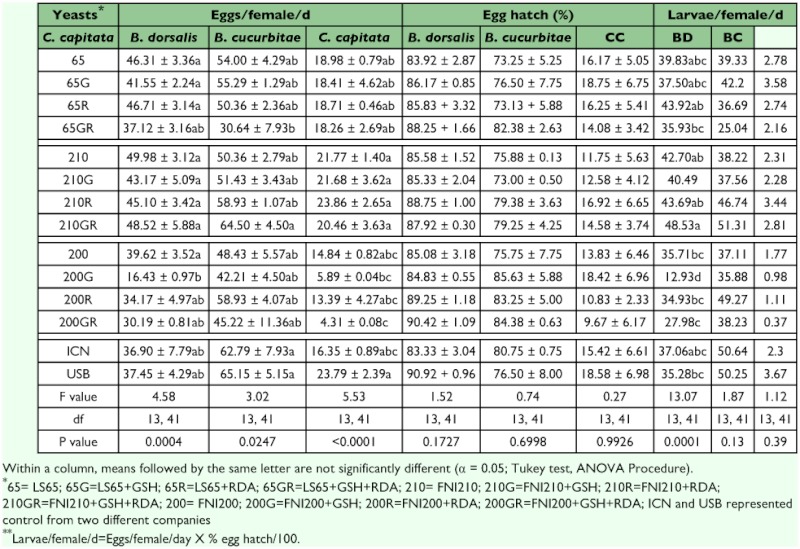
Evaluation of 14 yeasts in fruit fly adult diet for *C. capitata* (CC), *B. dorsalis* (BD), and *B. cucurbitae* (BC) on egg production, egg hatch, and estimated larvae/female/day

### Yeast evaluation

The order of yeast evaluation in this study was performed as described below:

(1) Four yeasts with addition of wheat germ oil (1% of water volume) (FNILS65W, FNI200W, FNI210W, and KW) and Korea yeast (K) without wheat germ oil were first screened based on the parameters of percent pupal recovery, larval duration, pupal weight, percent adult emergence, percent fliers, mating, egg production, peak egging period, and egg hatch as described in Chang et al. 2006. (2) Those with the best performance (A, B) from (1) were further combined with GSH (G) or RDA (R) or both (GR) to see any enhancement. (3) The two best performers again (C, D) were compared with T with or without wheat germ oil (TW, T) to determine the most cost effective yeast. (4) The best performer (E) was then compared to LBI2240, LBI2240W, LBI2240G, LBI2240GW, LBI2240FNILS65W, and LBI2240FNILS65. Yeasts or their combinations that performed the best (F) were selected for mass rearing diet formulation for oriental fruit fly larvae. The yeast evaluation can be diagrammed as follows:
  - FNI200W, FNILS65W, FNI210W, KW, K (the two best = A, B)
  - AW, AGW, ARW, AGRW, BW, BGW, BRW, BGRW (the two best = C, D)
  - C, CW, D, DW, T TW (the best one = E)
  - LBI2240, LBI2240W, LBI2240G, LBI2240GW, LBI2240FNILS65W, LBI2240FNILS65, E (the best one = F)

### Data and statistical analysis

The criteria for evaluation of the liquid diet and its delivery system were larval duration, pupal recovery (number of pupae produced from number of seeded eggs; expressed as percent), pupal weight, adult eclosion, mating, percent of adults that could fly, egg production of subsequent generation, and egg hatch as described by Chang et al. (2006) and FAO/IAEA (1998; 2003). Data are presented as mean values ± SE (standard errors) and were obtained from at least four batches for each treatment. Differences among diets were determined by analysis of variance (ANOVA), and means were separated using a Tukey's test at α=0.05 (SAS Institute 2002).

**Table 3.**
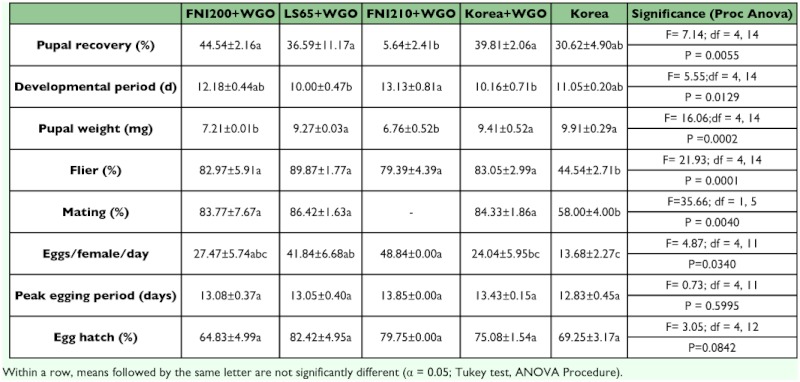
Screening of FNI200, LS65, FNI210, and Korea yeast on *B. dorsdis* larval liquid diet

## Results and Discussion

### Adult diets

As shown in Table 2, medfly produced the most eggs/female/day when fed yeasts FNILS65, FNILS65G, FNILS65R, FNI210, FNI210G, FNI210R, FNI210GR, FNI200. The addition of G, R, or GR did not increase egg output. *B. dorsalis* was most productive on FNI210GR and the ICN control diet. *B. curcurbitae* were most productive on FNI210 regardless of additional nutrients. This species also did well on the USB control diet. There were no significant differences in egg hatch regardless of the type of yeast in the diet of any of these fruit flies. The number of larvae/female/day (number of eggs × % hatch) for *C. capitata* was significantly greater than on the FNI210GR diet. There were no significant differences in larval production for *B. dorsalis* or *B. curcurbitae*. Thus the best diets were FNI210GR for *C. capitata*, FNI210GR or ICN for *B. dorsalis*, and FNI210 or USB for *B. curcurbitae*.

### Liquid diets

The following steps are how we identified the most costeffective yeast for a liquid diet for the oriental fruit fly, *B. dorsalis*.

To identify the best potential yeasts from our available stock, which included FNI200, FNILS65, FNI210. These yeasts were incorporated in the standard liquid diet with wheat germ oil because our previous work showed that addition of wheat germ oil to a liquid diet would enhance performance (Chang and Vargas 2007). Preliminary tests showed that FNI200, FNILS65, and Korea yeasts were the most promising based on numbers of pupa produced and the flight and mating ability of adults.

Pupal recovery from larvae reared in FNI200W, FNILS65W, KW or K diet was not different among them and was significant higher than those reared in FNI210W diet. Adult flight and mating from larvae reared in FNI200W, FNILS65W, or KW diet were also significantly higher than those from those reared in K diet. There were no data on mating from the FNI210W diet because there were not enough adults to perform the mating test. Among the diets FNI200W, FNILS65W, FNI210W, KW, and K, the diet with FNI210W yielded the fewest pupae. Larvae reared in diets with FNILS65 and KW developed faster than FNI200W and FNI210W. More adults emerged from larvae reared in FNI200W and FNILS65W diet than those from diet FNI210W, KW, and K although there were no significant differences among these three. Egg production from larvae reared in diet with FNI210W, FNI200W, or FNILS65W produced the highest number of eggs although there were not significant differences among FNI200W, FNI210W and FNILS65W. However, egg production from diets with K was low. There were no differences in egg hatch between treatments (Table 3).

**Table 4.**
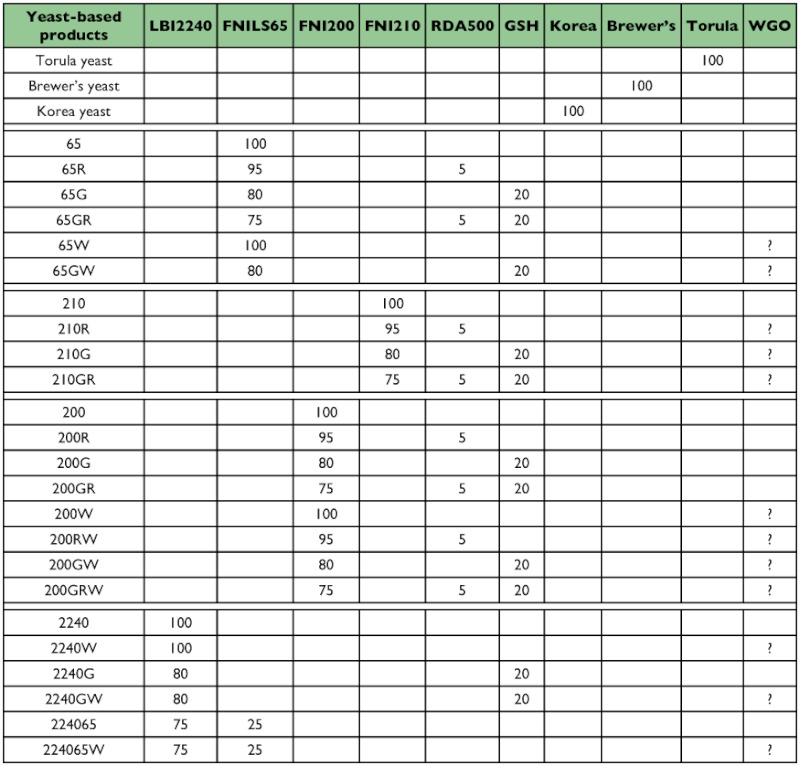
Relative proportion of the different proteins sources used for each liquid diet tested

These results indicate that there is a similarity in nutrients needed by adults and larvae. Both adults and larvae of fruit flies fed on FNI210W diet are able to produce more eggs than those fed on other diets. Based on the results above, FNI200W, FNILS65W, and KW were promising. KW was not examined further because it availability and its solidified character. Therefore, FNI200 and FNILS65 were selected for further evaluation.

Glutamine enriched yeast G and/or high vitamin enriched yeast R were incorporated into diets with FNI200 and FNILS65. Diets named FNI200W, FNI200RW, FNI200GW, FNI200GRW, FNILS65W, FNILS65RW, FNILS65GW, FNILS65GRW were derived from the combination of FNI200 or FNILS65 with G in a ratio of 80:20 or RDA in a ratio of 95:5, or both (GR), respectively (see Table 4 for details of these combined diets). Among these eight diets, larvae reared in diets with FNI200GW, FNI200GRW, FNILS65GW, and FNILS65GRW have a similar performance, and produced the highest percentage of pupae. Diets with FNI200RW and FNILS65RW were in the second rank, and FNI200W and FNILS65W produced significantly lower percentage of pupae than those from others. This suggests that GSH, glutamine enriched yeast, was able to enhance the performance while high vitamin enriched yeast RDA500 did not affect performance. Larvae reared in diets with FNILS65 series especially FNILS65GW and FNILS65GRW developed faster and produced heavier pupae than those from diets with FNI200 series, although they were not significantly different in other parameters. Diets with FNI200GW and FNILS65GW were selected for further evaluation because they performed the best (Table 5).

To determine the most cost-effective yeast to be used in the liquid diet, diets with FNI200G, FNI200GW, FNILS65G, FNILS65GW, TW, and T were evaluated and compared to the control diet (traditional standard diet) (Tanaka et al 1969). Torula yeast (T) was included as it is cheaper and often available locally. As shown in Table 6, larvae reared in both diets either with FNI200GW or FNILS65GW yielded a similar percentage of pupae as the control diet while the torula diets (T, TW) produced the lowest percent pupae. However, larvae reared in diet with TW developed in the shortest time in comparison to those from other diets. Larval developmental time from diets with FNILS65 series are relatively shorter than those from FNI200 series but longer than torula series (Table 6) and it is about one day longer in comparison to the control diet. Larvae reared in all these diets were identical in weight except FNI200GW in which they were slightly lighter. There was no significant difference in adult eclosion among all test diets except FNI200G. The number of adult fliers and number of mating adults produced from a liquid diet with FNI200G or FNILS65G without wheat germ oil were significantly lower than those from a diet with FNI200GW, FNILS65GW, T, TW, or the mill feed control diet while there was no significant difference between the latter diets. Egg production and egg hatch from adults reared from control diet or diets with wheat germ oil were significantly higher than those from reared in diets without wheat germ oil. Overall, the FNILS65GW diet performed the best suggested that it may be because hydrolyzed yeast contain more minerals especially phosphorous and potassium. It is still questionable whether minersals are the cause of these differences. It will not be resolved until a completely chemically defined diet is developed. Neverthless, hydrolyzed yeast is more costly than whole cell yeast..

**Table 5.**
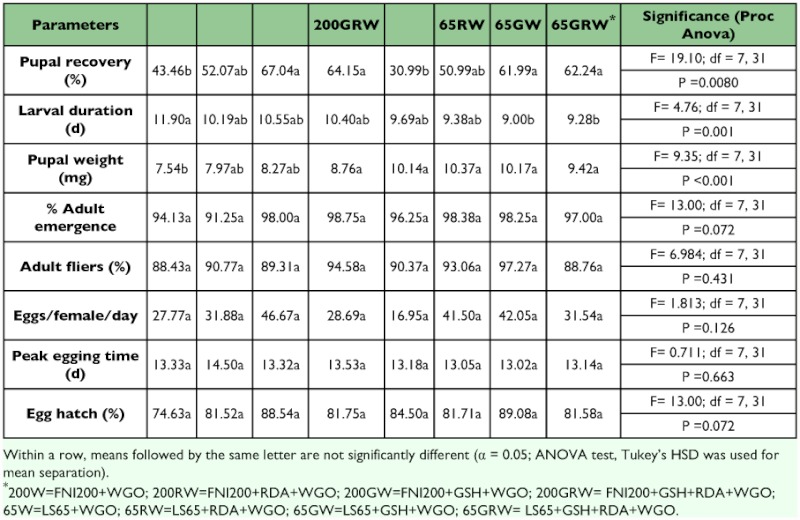
Effect of RDA and/or GSH on fruit fly performance when RDA and/or GSH were incorporated into liquid larval diet

**Table 6.**
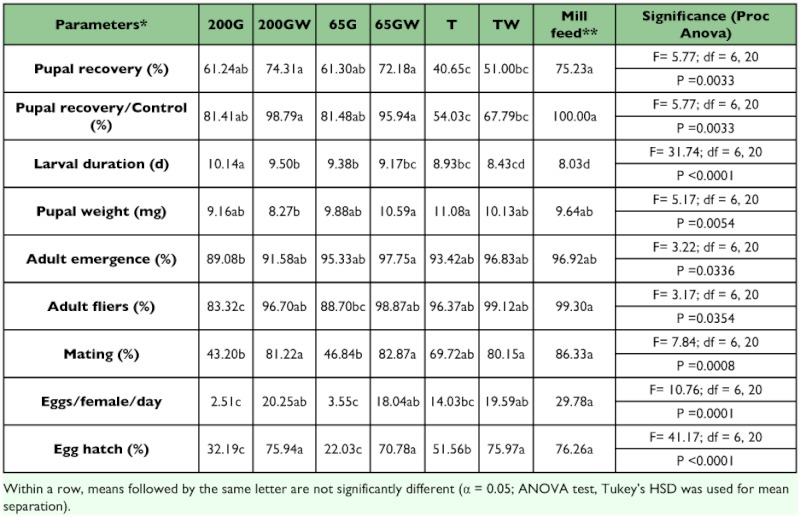
Comparison of fruit fly performance from rearing *B. dorsalis* in diet with FNI200, LS65, and torula yeast

**Table 7.**
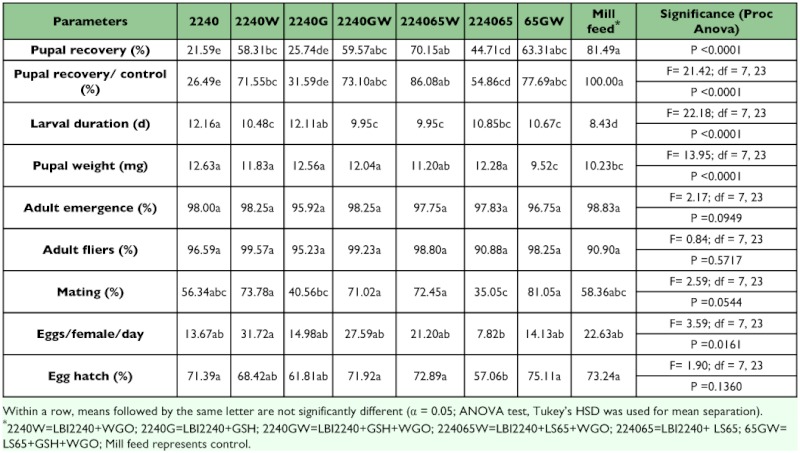
Comparison of fruit fly performance after rearing *B. dorsalis* larvae in LBI2240, LBI2240 and LS65 mix, or LS65 diet

A novel, cheaper whole cell yeast (LBI2240) was cultured and provided by Lallemand Inc. This whole cell yeast has been evaluated in medfly liquid larval diet (Chang et al. 2007). It costs one fifth of the hydrolyzed yeast FNILS65. Therefore, diets with yeast LBI2240 series (LBI2240, LBI2240W, LBI2240G, LBI2240GW, LBI2240FNILS65, and LBI2240FNILS65W) were compared with FNILS65GW and control diet. Pupal recoveries from larvae reared in diets with LBI2240GW, LBI2240FNILS65W, and FNILS65GW are the same as those from the control diet. Larval duration of those reared with LBI2240GW or LBI2240FNILS65W developed faster than others although it was still about 1.5 days slower than those from the control diet. This delay can be compensated with rearing fruit fly at a higher temperature (unpublished observations). All pupae from larvae reared in diet with LBI2240 series were either heavier or equal to pupae from the control diet. There was no significant difference among all diets in either adult emergence or adult flying ability. Adult flies from larvae reared in diets with wheat germ oil (2240W, 2240GW, 2240FNILS65W, 65GW) exhibited better mating ability although there were no statistically significant differences from the control. Egg production and egg hatch were not significantly different among all diets tested except those reared in diet with LBI2240FNILS65 and this is probably due to the lack of wheat germ oil in the diet (Table 7).

In conclusion, we select and recommend LBI2240 + FNILS65W (3:1) instead of FNILS65GW as the most cost-effective yeast for oriental fruit fly liquid larval diet in our study. How and why these two yeasts are better than others will not be clear until we understand the functional relationships between diet components and insect performance. Therefore, basic and applied insect nutritional research is needed. However, we highly recommend using local yeast if there are yeasts with similar nutrients that are available locally. However, an evaluation similar to that done here would be very important. For adult diets, both FNILS65 and FNI210 series are equally good for all three species but FNI200G and FNI200 GR should be avoided for both *C. capitata* and *B. curcurbitae*.
